# Impact of Perinatal Different Intrauterine Environments on Child Growth and Development: Planning and Baseline Data for a Cohort Study

**DOI:** 10.2196/12970

**Published:** 2019-11-12

**Authors:** Isabel Cristina Ribas Werlang, Juliana Rombaldi Bernardi, Marina Nunes, Thiago Beltram Marcelino, Vera Lucia Bosa, Mariana Bohns Michalowski, Clécio Homrich da Silva, Marcelo Zubaran Goldani

**Affiliations:** 1 Laboratório de Pediatria Translacional Centro de Pesquisa Experimental Hospital de Clínicas de Porto Alegre Porto Alegre Brazil; 2 Programa de Pós-Graduação em Saúde da Criança e do Adolescente Departamento de Pediatria Universidade Federal do Rio Grande do Sul Porto Alegre Brazil; 3 Programa de Pós Graduação em Alimentação, Nutrição e Saúde Departamento de Nutrição Universidade Federal do Rio Grande do Sul Porto Alegre Brazil

**Keywords:** observational study, growth, development, fetal development child health, maternal health

## Abstract

**Background:**

Several studies have shown that exposure of the fetus and newborn to prenatal and perinatal events, respectively, may influence the health outcomes of the child throughout their life cycle.

**Objective:**

This study aimed to increase the knowledge on the impact of different intrauterine environments on child growth and development, as we know that pregnancy and early years are a window of opportunity for health promotion and prevention interventions of diseases.

**Methods:**

The recruitment occurred 24 to 48 hours after delivery and involved mothers and their newborns in 2 public hospitals in Porto Alegre, Brazil, from December 2011 to January 2016. The mothers-newborns dyads were allocated to 5 groups: diabetes mellitus, mothers with a clinical diagnosis of diabetes; systemic arterial hypertension (SAH), mothers with a clinical diagnosis of systematic arterial hypertensive disease during pregnancy; maternal smoking, mothers who smoked at any moment of gestation; small for gestational age (SGA), mothers with SGA newborns because of intrauterine growth restriction; and control, mothers without the clinical characteristics previously mentioned. Several protocols and anthropometric measurements were applied in the interviews at immediate postpartum and 7 and 15 days and 1, 3, and 6 months after birth. For this study, we analyzed only data collected during postpartum interviews. The statistical analyses were performed using Pearson chi-square test, Mann-Whitney test, or Kruskal-Wallis test with Dunn post hoc. The significance level was set at 5%. The Hospital Ethics and Research Committees approved the study.

**Results:**

Of the 485 eligible mothers-newborns dyads, 400 agreed to participate (82.5%, 400/485). As expected, newborns from the SGA group had significantly lower birth weight, smaller stature, and lower cephalic perimeter (*P*<.001). This group also had the highest percentage of primiparous women in comparison with other groups (*P*=.005) except for control. Mothers from the SAH group had the highest mean age, the highest percentage of cesarean sections, and presented greater gestational weight gain.

**Conclusions:**

In this study, we describe the planning and structure for the systematic follow-up of mother-newborn dyads in the first 6 months after birth, considering the important demographic and epidemiological transition scenario in Brazil. The results of this prospective longitudinal study may provide a better understanding of the causal mechanisms involved in health and life course disease related to different adverse intrauterine environments.

## Introduction

### Background

Several studies have described the influence of intra- and extrauterine environment changes on human growth and development, leading to a particular health-disease pattern during life course [1,2]. Metabolic variation during the gestational period can provide an unfavorable environment to fetal growth, triggering structural and functional adaptations with permanent effects on organs and tissues of the individual [3,4].

Fetal exposure to high glucose concentrations can lead to changes in neuroendocrine metabolism, acting on metabolic programming and potentially contributing to the development of type 2 diabetes mellitus (DM) in adulthood [5]. Intrauterine hypoxia associated with hypertension during pregnancy also contributes to perinatal morbidity and mortality, and to the possibility of cognitive deficit in offspring, exemplifying the concept of transgenerational risk of cardiovascular disease [6,7].

In the same way, maternal smoking (MS) causes changes in immunity and lung function during the neonatal period [8]. Other effects include attention deficit hyperactivity disorder, motor function problems, cognitive deficits [9], increased incidence of asthma [10], and increased risk of developing obstructive pulmonary chronic disease [11].

On the contrary, small for gestational age (SGA) infants per se have been associated with changes in the metabolism of fetal glucose and insulin homeostasis, altering metabolic mechanisms essential for short-term and long-term postnatal health and disease outcomes [12].

Thus, the analysis of the impact of those adverse intrauterine environments mentioned on infant growth and development can contribute to new insights on mechanisms related to health and disease, and in the development of early interventions, which are decisive for chronic diseases prevention [4].

### Objectives

The impact of perinatal environment variations on health of the newborn at first 6 months of life (IVAPSA) study aimed to identify the effects of the intrauterine and perinatal environmental variations on infant growth and development during the first 6 months of life. The study has offered a remarkable approach to the theme and increased the knowledge upon the impact of different intrauterine environments on health and disease outcomes during childhood and adulthood.

## Methods

### Design and Population

The participant recruitment occurred from December 2011 to January 2016 in 2 public hospitals in Porto Alegre, Brazil: Hospital de Clínicas de Porto Alegre (HCPA) and Grupo Hospitalar Conceição (GHC). We invited mothers delivering between 37 and 42 weeks of gestation to participate; they should fit into the following groups:

- DM: clinical diagnosis of diabetes, considering any disease classification (gestational, type 1 and 2).
- Systemic arterial hypertension (SAH): presence of hypertensive disease during pregnancy (chronic, preeclampsia, eclampsia and overlapping diseases).
- MS: mothers who confirmed in a specific questionnaire that had smoked at any moment of gestation, regardless of the number of cigarettes.
- SGA: mothers of SGA newborns–categories below the fifth percentile of the Alexander curve [13] because of intrauterine growth restriction.
- Control: group without the clinical characteristics previously mentioned. Mothers without confounding comorbidities were prioritized in the recruitment, that is, those without the coexistence of other factors previously mentioned.

Those excluded from the analysis were the following participants:

- HIV positive: intrauterine exposure to the virus, use of antiretroviral drugs, and contraindication to breastfeeding.
- Twin or preterm newborns: born with low birth weight, may present greater neonatal vulnerability and also develop catch-up growth during the first year of life.
- Newborns with congenital malformations: more vulnerable to diseases and are likely to require neonatal hospitalization.
- Newborns who require early hospitalization: generally become more vulnerable and adversely affect their growth and development.

The definition of these exclusion criteria was based on the fact that they possibly contribute to changes in the growth and development of the newborn and infant within the first 6 months of life and affect their future health and disease outcomes.

The Research and Ethics Committees of the HCPA (protocol No. 11/0097) and the GHC (protocol No. 11/027) approved our study. All participants were recruited after written informed consent. All methods were performed in accordance with the latest current guidelines and regulations of the National Health Council of the Brazilian Ministry of Health (resolutions No. 466/2012 and No. 580/2018).

### Assessment of Participant Characteristics

Mothers-newborns dyads were recruited 24 to 48 hours after delivery. The follow-up was conducted at 7 and 15 days and at 1, 3, and 6 months of the infant’s life (Table 1). During the follow-up, through various protocols, information was obtained on (1) the family: demographic and socioeconomic characteristics; (2) the mother: age, schooling, profession, stress, domestic violence, postpartum depression, and confidence; (3) during gestation: prenatal care and parity; (4) the perinatal period: type of delivery, gestational age, Apgar score, gender, weight, height, and cephalic perimeter of newborn; (5) the infant: growth, development, and sleep; (6) maternal and infant feeding; and (7) maternal and infant anthropometric measures. Mothers agreed to provide breast milk in the postpartum period and at 1 month after birth. Saliva from mothers and their newborns were collected in the postpartum period for DNA extraction. More detailed information about the assessments, protocols, and the IVAPSA study can also be verified in a previous publication [14].

Specifically, for this study, we analyzed only data collected during postpartum recruitment. The maternal variables evaluated were age (years), schooling (years), number of previous pregnancies and parity, pregestational body mass index (BMI), and weight gain during pregnancy (kg). The gestational assistance variables used were delivery mode and number of prenatal consultations. Newborn variables included gender, weight (g), length (cm), cephalic perimeter (cm), and Apgar index.

In addition, we promoted an active search in social networks (basic health units, schools, markets, and commercial establishments) and used the post office system to access the most distant or unsafe neighborhoods and interviews previously scheduled by telephone on weekends and holidays.

**Table 1:** Variables, protocols, and collected sample performed in the study of impact of perinatal environment variations on health of the newborn at first 6 months of life (2011-2016).

Interviews		Postpartum	7 days	15 days	1 month	3 months	6 months
Location		Hospital	Home care	CRC^a^	CRC	Home care	CRC
**Variables**							
	Gestational	X	—^b^	—	—	—	—
	Demographic and socioeconomic characteristics	X	—	—	—	—	—
	Perinatal	X	—	—	—	—	—
	Environmental	X	X	X	X	X	X
	Maternal, newborn, and infant feeding practices	—	X	X	X	X	X
**Protocols**							
	Maternal violence	—	—	—	X	—	X
	Maternal care	—	X	X	—	—	—
	Maternal confidence	—	—	X	—	—	—
	Maternal perception	—	—	X	—	—	—
	Maternal perceived stress	—	—	—	X	—	—
	Postpartum depression	—	—	—	X	X	X
	Breastfeeding observation	X	—	—	—	—	—
	Parental care	—	—	—	—	X	—
	Child development	—	X	—	X	X	X
	Child’s sleep	—	—	—	X	X	X
	Mother-child bond	—	—	—	—	X	X
**Collected samples**							
	Saliva	X	—	—	—	—	—
	Breast milk	X	—	—	X	—	—
	Anthropometry	—	X	X	X	X	X

^a^CRC: Clinical Research Center, Hospital de Clínicas de Porto Alegre.

^b^Data were not collected.

### Statistical Analysis

Database processing and analysis were performed using SPSS software (version 18.0; SPSS Inc, Chicago, IL). The descriptive statistical analysis verified the absolute and relative frequency of categorical variables. For continuous variables, central tendency measurements were obtained according to the variable distribution. The Pearson chi-square test or Mann-Whitney test was used to analyze the differences between the group of participating mothers and the refusals. To evaluate the differences between the study groups, Pearson chi-square test or Kruskal-Wallis test with Dunn post hoc was used.

## Results

### Recruitment

Before the recruitment, from September to December 2011, a pilot study was conducted, which covered 17 mothers-newborns dyads. Finally, the study presented a total of 485 eligible mothers-newborns from which 85 were refusals, leading to a total sample of 400 pairs (82.5%, 400/485). The characteristics of participants and refusals in the recruitment are presented in Table 2.

Considering differences between participants and nonparticipants, there was a significant distinction for delivery mode (higher incidence of vaginal delivery among mothers who agreed to participate, *P*<.001) and for the number of pregnancies (greater number of pregnancies among participating mothers, *P*<.001). On the contrary, it was observed that other maternal and newborn characteristics were similar.

Of mothers who participated in the postnatal study (n=400), 58.8% (235/400) were interviewed at 7 days, 66.0% (264/400) at 15 days, 66.8% (267/400) with 1 month, 67.0% (268/400) with 3 months, and 58.8% (235/400) with 6 months of life of the child. The profile of refusals, losses of tracking, and data recovered by each interview by groups can be verified in Table 3.

**Table 2:** Characteristics of mother-newborn dyads who accepted and refused to participate in the initial study interview (2011-2016).

Variables		Participants (N^a^=400)	Refusals (N^a^=85)	*P* value
**Mother**				
	Age (years), mean (SD)	26.15 (6.60)	27.90 (7.01)	.30
	Education (years), mean (SD)	9.26 (2.73)	10.00 (7.10)	.66
	White, n (%)	209 (54.9)	53 (62)	.21
	Vaginal delivery, n (%)	248 (65.1)	32 (35)	<.001^b^
	Number of pregnancies, mean (SD)	3.10 (1.43)	2.07 (1.46)	<.001^c^
**Newborn**				
	Weight (g), mean (SD)	3234.01 (499.85)	3287.23 (497.38)	.46
	Length (cm), mean (SD)	48.56 (2.25)	48.69 (2.05)	.78
	Cephalic perimeter (cm), mean (SD)	33.79 (1.54)	34.10 (1.36)	.07
	Apgar 1st min, mean (SD)	8.39 (1.33)	8.39 (1.33)	.92
	Apgar 5th min, mean (SD)	9.45 (0.63)	9.48 (0.80)	.25

^a^N refers to the entire population under study.

^b^Pearson chi-square test.

^c^Mann Whitney test.

**Table 3:** Frequency of interviewed participants, recovered data, losses of follow-up, and refusals during the study (2011-2016).

Interviews		DM^a^	SAH^b^	MS^c^	SGA^d^	Control	Total
**7 days, n (%)**							
	Conducted	46 (59)	24 (64)	49 (56)	26 (70)	90 (55)	235 (58.8)
	Recovered data	13 (16)	9 (24)	17 (19)	7 (18)	29 (18)	75 (18)
	Losses	17 (21)	4 (10)	20 (23)	4 (10)	38 (23)	83 (21)
	Refusals	2 (2)	0 (0)	1 (1)	0 (0)	4 (2)	7 (2)
	Total	78 (19)	37 (9)	87 (22)	37 (9)	161 (40.3)	400 (100.0)
**15 days, n (%)**							
	Conducted	54 (69)	30 (81)	53 (60)	25 (67)	102 (63.4)	264 (66.0)
	Recovered data	0 (0)	1 (3)	4 (4)	1 (3)	8 (5)	14 (3)
	Losses	21 (26)	5 (13)	27 (31)	11 (29)	50 (31)	114 (28.5)
	Refusals	3 (4)	1 (3)	3 (3)	0 (0)	1 (16)	8 (2)
	Total	78 (19)	37 (9)	87 (22)	37 (9)	161 (40.3)	400 (100.0)
**1 month, n (%)**							
	Conducted	50 (64)	26 (70)	55 (63)	27 (73)	109 (67.7)	267 (66.8)
	Recovered data	0 (0)	1 (3)	2 (2)	0 (0)	4 (2)	7 (2)
	Losses	19 (24)	9 (24)	21 (24)	10 (27)	36 (22)	95 (23)
	Refusals	9 (11)	1 (3)	9 (10)	0 (0)	12 (7)	31 (8)
	Total	78 (19)	37 (9)	87 (22)	37 (9)	161 (40.3)	400 (100.0)
**3 months, n (%)**							
	Conducted	51 (65)	26 (70)	58 (66)	26 (70)	107 (66.5)	268 (67.0)
	Recovered data	1 (1)	1 (3)	0 (0)	0 (0)	1 (1)	3 (1)
	Losses	19 (24)	8 (22)	20 (23)	10 (27)	35 (22)	92 (23)
	Refusals	7 (9)	2 (5)	9 (10)	1 (3)	18 (11)	37 (9)
	Total	78 (19)	37 (9)	87 (22)	37 (9)	161 (40.3)	400 (100.0)
**6 months, n (%)**							
	Conducted	48 (61)	25 (68)	43 (49)	25 (68)	94 (58)	235 (58.8)
	Losses	23 (29)	10 (27)	32 (37)	10 (27)	47 (29)	122 (30.5)
	Refusals	7 (9)	2 (5)	12 (14)	2 (5)	20 (12)	43 (11)
	Total	78 (19)	37 (9)	87 (22)	37 (9)	161 (40.3)	400 (100.0)

^a^DM: diabetes mellitus.

^b^SAH: systemic arterial hypertension.

^c^MS: maternal smoking.

^d^SGA: small for gestational age.

### Analysis of Variables From Newborns

As expected, newborns from the SGA group had significantly low birth weight, height, and head circumference (*P*<.001). Despite the different intrauterine environments, there was no statistically significant difference in the Apgar 1 and 5 indexes, and in the gender distribution (Table 4).

### Analysis of Maternal and Health Care Variables

Mothers from the SAH group had the highest median age and a higher percentage of cesareans. In addition, these mothers presented greater gestational weight gain. Mothers from the DM group had the highest values of pregestational BMI. The educational level of women smokers was statistically lower when compared with DM and control (*P*=.005). In addition, the MS group presented a higher percentage of mothers who did less than 7 prenatal consultations. Mothers of the SGA group had the lowest median age, and a lower median pregestational BMI and lower weight gain during gestation. This group also had the highest percentage of primiparous women compared with other groups (*P*=.005), with the exception of control (Table 4).

**Table 4:** Characteristics of the newborn and mothers related to study groups.

Variables		DM^a^ (N^b^=78)	SAH^c^ (N^b^=37)	MS^d^ (N^b^=87)	SGA^e^ (N^b^=37)	Control (N^b^=161)	*P* value
**Newborn**							
	Gender (female), n (%)	39 (50)	19 (51)	43 (49)	22 (59)	89 (55.2)	.78
	Weight at birth (g), median (range)	3416.60 (2475 to 4760)	3182.84 (2125 to 4630)	3114.88 (2260 to 4000)	2517.46 (2090 to 2760)	3380.59 (2400 to 4965)	<.001^f^
	Length at birth (cm), median (range)	48.95 (45 to 53)	48.08 (44 to 53)	48.04 (42 to 53)	46.07 (42 to 49.5)	49.33 (43.5 to 59)	<.001^f^
	Cephalic perimeter (cm), median (range)	34.12 (31 to 37)	34.00 (32 to 36.5)	33.73 (31 to 37)	31.92 (30 to 34.5)	34.04 (30.5 to 38)	<.001^f^
	Apgar 1st min, median (range)	8.31 (3 to 10)	8.29 (3 to 10)	8.51 (2 to 10)	8.35 (4 to 10)	8.37 (3 to 10)	.61
	Apgar 5th min, median (range)	9.29 (7 to 10)	9.49 (8 to 10)	9.51 (7 to 10)	9.58 (9 to 10)	9.43 (7 to 10)	.19
**Mothers**							
	Age (years), median (range)	28.15 (17 to 40)	29.05 (14 to 42)	24.60 (16 to 39)	23.84 (13 to 41)	25.73 (15 to 42)	<.001^f^
	Maternal education (years), median (range)	9.62 (1 to 17)	9.32 (3 to 17)	8.37 (3 to 15)	9.72 (5 to 15)	9.49 (0 to 17)	.005^f^
	Number of pregnancies, median (range)	3.37 (2 to 8)	3.08 (1 to 7)	3.06 (1 to 8)	2.69 (2 to 6)	3.05 (1 to 9)	.56
	Primiparous, n (%)	27 (35)	10 (27)	25 (29)	21 (57)	74 (45.9)	.005^g^
	Number prenatal consultations, n (%), <7 consultations	13 (17)	5 (13)	49 (56)	14 (38)	50 (31.0)	<.001^g^
	Type of delivery, n (%) vaginal	46 (59)	11 (30)	65 (75)	24 (65)	114 (70.8)	<.001^g^
	Pregestational BMI (kg/m^2^), median (range)	28.49 (19.8 to 55.9)	27.92 (18.3 to 41.5)	24.79 (15.4 to 43.3)	22.78 (16.9 to 35.3)	24.51 (18.0 to 41.6)	<.001^f^
	Gestational weight gain (kg), median (range)	13.05 (−3 to 36.0)	16.42 (0 to 31.5)	13.81 (−2.6 to 30.8)	11.48 (−3.0 to 25.8)	13.64 (−9.0 to 33.5)	.02^f^

^a^DM: diabetes mellitus.

^b^N: refers to the entire population under study.

^c^SAH: systemic arterial hypertension.

^d^MS: maternal smoking.

^e^SGA: small for gestational age.

^f^Kruskal-Wallis test with Dunn post hoc.

^g^Pearson chi-square test.

## Discussion

### Principal Findings

This study presented an original methodological prospective longitudinal design [14], focusing on maternal and infant clinical trajectory during the first 6 months of life. It was conducted in a city with 1.5 million inhabitants in a vast geographic area. The follow-up strategy performed a high number of interventions in a short period, leading to several methodological challenges [14]. Initially, a pilot study was conducted to investigate the feasibility of the research and to consolidate the methodology and logistics in this study. Strategies were applied to improve the mothers’ adherence throughout the study, such as home visits. Interviews at 7 and 15 days and at 3 months were chosen because they do not include protocols that are difficult to execute or need specific devices, whereas postpartum 1 month and 6 months were carried out at the Clinical Research Center.

Usually, the follow-up is planned with larger intervals between interventions in the classical longitudinal studies carried out in Brazil. In the Pelotas cohort, the steps were at 3, 12, 24, and 48 months of age of the child [15]. In the IVAPSA study, the purpose was to establish several interventions in closer intervals up to 6 months of age [14]. Some protocols related to the mother’s psychological parameters and the child’s gross motor development contributed to the establishment of the selected periods [16,17].

Refusals to participate and dropouts and follow-up lost are expected in all cohort studies [18]. However, they can be minimized by consolidating an adequate relationship between the participants and the researchers, making the first contact very decisive for follow-up. In this study, most of the characteristics of the mothers and their newborns who refused to participate did not differ from those who consented to.

As expected, the SGA newborn group showed lower birth weight, length, and cephalic perimeter [19,20]. In a study published by our group, we found that, in addition to these characteristics, mothers from the SGA group have shown low levels of leptin and insulin in the transition from colostrum to mature milk [21]. This finding can be associated with the rapid weight gain of these newborns in the first month of life. Another publication by our research team has shown that newborns from the SGA group had a greater impact on the growth trajectory in the first 6 months in comparison with MS and control groups, even considering factors such as MS or diet [22].

The mothers from the DM group had a higher pregestational BMI and delivered heavier newborns in relation to the other intrauterine groups. This result corroborates previous findings that also detected an association between pregestational BMI greater than 25 kg/m² and fetal macrosomia [23,24]. As demonstrated in other studies, lower birth weight medians were observed in the HAS, MS, and SGA groups [6,25-27]. All these intrauterine environments can be related to reduction in the placental perfusion, owing to increased blood pressure and constriction vase, causing a deficit in fetal growth [25,27]. Regardless of this reduction in the placental perfusion that can also affect birth conditions [27], the Apgar index was similar among the groups in this study. Besides, some factors have been associated with the birth of SGA children, such as preterm delivery, short maternal stature, mother’s low weight, maternal age, and unfavorable socioeconomic conditions [28]. Factors such as age or pregestational BMI were not different in the SGA group compared with the control group, in which children were not SGA.

The mothers of the SAH group had older age, higher median for gestational weight gain, and high pregestational BMI, results that were also observed by other authors [29]. According to the guidelines of the Institute of Medicine from the United States, the recommendation is that women with a BMI between 25 and 29.9 kg/m² should have gestational weight gain between 7 to 11.5 kg [30].

Mothers from the smoking group had lower educational level and lower total number of prenatal consultations, demonstrating an environment of social vulnerability. The pregnant woman’s knowledge of her health condition (DM or SAH) may determine the demand for more frequent care than the others. A study published with data from this cohort, using MS and control groups, found that the number of prenatal consultations was negatively influenced by MS during pregnancy and by the number of children, along with a positive correlation regarding maternal age [31]. In the general analysis of groups, a higher frequency of more than 7 visits (67%) was observed, which corroborates with data observed in a previous study, evidencing an increase in prenatal coverage in Porto Alegre [32]. Currently, the World Health Organization recommends a minimum of 8 prenatal consultations to reduce perinatal mortality and improve the experience of care for women [33]. In the southern region of Brazil, the number of births from pregnant women with more than 6 prenatal visits between the years 2000 and 2010 increased from 53.2% to 75.5%, and the demand for prenatal care was higher among women over 25 years of age [34,35].

Vaginal delivery was performed in most of the samples, except in the SAH group owing to obstetric peculiarities that usually require surgical delivery. Referring to the number of previous pregnancies, comparative data between the years 2004 and 2014 showed a decrease in the total fertility rate in Brazil from 2.14 to 1.74 [36]. In this study, it was found that all groups had a higher number of gestations when compared with the last Brazilian average.

### Limitations

The random distribution of the participants’ home, the vast distance traveled by the research team, and the high level of insecurity in some areas were some difficulties to conduct a longitudinal study in a large city in Brazil, different from classic cohort studies in small or medium-size cities such as Pelotas [15] and Ribeirão Preto [37]. Other important issues include obtaining financial support for the transportation of participants and researchers, permanent training of the research team, high frequency of changes at participants’ addresses, and duration of the interview because of many questionnaires, ranging from 1 to 2.5 hours. Regarding the collection of biological materials, the main difficulty was the extraction of breast milk (colostrum) during the postpartum visit.

### Strengths

In this study, the main strengths were the planning and structuring of a birth follow-up, considering the scenario of demographic and epidemiological transition in Brazil [36,38]. This process is characterized by an intense change in the pattern of health and disease throughout the population with an increased number of pregnant women with high obstetric risk. The adoption of a convenience sample from public hospitals and exclusion criteria were necessary to overcome difficulties in sample recruitment and allowed to control possible confounding such as gestational age, social class, and perinatal and prenatal intercurrences, facilitating the analysis of different outcomes between groups.

### Conclusions

Considering the influence of intrauterine environments on the health outcomes in children and adults, and the potential interventions during pregnancy and at the newborn’s first years of life, it is essential to understand the patterns of growth and development related to maternal clinical background. Therefore, this prospective longitudinal study with innovative design can bring new insights about causal mechanisms involved in health and illness of individual process and provides opportunities for public health promotion with prevention strategies.

## Abbreviations

BMI: body mass index
DM: diabetes mellitus
GHC: Grupo Hospitalar Conceição
HCPA: Hospital de Clínicas de Porto Alegre
IVAPSA: impact of perinatal environment variations on health of the newborn at first 6 months of life
MS: maternal smoking
SAH: systemic arterial hypertension
SGA: small for gestational age

## References

[1] Calkins K, Devaskar SU (2011). Fetal origins of adult disease. Curr Probl Pediatr Adolesc Health Care.

[2] Simeoni U, Armengaud J, Siddeek B, Tolsa J (2018). Perinatal origins of adult disease. Neonatology.

[3] Barker DJ, Eriksson JG, Forsén T, Osmond C (2002). Fetal origins of adult disease: strength of effects and biological basis. Int J Epidemiol.

[4] Gluckman PD, Hanson MA (2007). Developmental plasticity and human disease: research directions. J Intern Med.

[5] Chandler-Laney P (2010). Possible link between mothers’ high blood sugar during pregnancy and children’s reduced insulin sensitivity?. Pediatric Heal.

[6] Kvehaugen AS, Dechend R, Ramstad HB, Troisi R, Fugelseth D, Staff AC (2011). Endothelial function and circulating biomarkers are disturbed in women and children after preeclampsia. Hypertension.

[7] Pinheiro TV, Brunetto S, Ramos JG, Bernardi JR, Goldani MZ (2016). Hypertensive disorders during pregnancy and health outcomes in the offspring: a systematic review. J Dev Orig Health Dis.

[8] Aagaard-Tillery KM, Porter TF, Lane RH, Varner MW, Lacoursiere DY (2008). In utero tobacco exposure is associated with modified effects of maternal factors on fetal growth. Am J Obstet Gynecol.

[9] Pagani LS (2014). Environmental tobacco smoke exposure and brain development: the case of attention deficit/hyperactivity disorder. Neurosci Biobehav Rev.

[10] Burke H, Leonardi-Bee J, Hashim A, Pine-Abata H, Chen Y, Cook DG, Britton JR, McKeever TM (2012). Prenatal and passive smoke exposure and incidence of asthma and wheeze: systematic review and meta-analysis. Pediatrics.

[11] Stocks J, Sonnappa S (2013). Early life influences on the development of chronic obstructive pulmonary disease. Ther Adv Respir Dis.

[12] Devaskar SU, Chu A (2016). Intrauterine growth restriction: hungry for an answer. Physiology (Bethesda).

[13] Alexander GR, Himes JH, Kaufman RB, Mor J, Kogan M (1996). A United States national reference for fetal growth. Obstet Gynecol.

[14] Bernardi JR, Ferreira CF, Nunes M, da Silva CH, Bosa VL, Silveira PP, Goldani MZ (2012). Impact of perinatal different intrauterine environments on child growth and development in the first six months of life--IVAPSA birth cohort: rationale, design, and methods. BMC Pregnancy Childbirth.

[15] Santos IS, Barros AJ, Matijasevich A, Domingues MR, Barros FC, Victora CG (2011). Cohort profile: the 2004 Pelotas (Brazil) birth cohort study. Int J Epidemiol.

[16] Pridham KF, Chang AS (1989). What being the parent of a new baby is like: revision of an instrument. Res Nurs Health.

[17] Liao PJ, Campbell SK (2004). Examination of the item structure of the Alberta infant motor scale. Pediatr Phys Ther.

[18] Coeli C, Faerstein E (2008). Estudos de coorte. Epidemiologia. Second edition.

[19] Dacaj R, Izetbegovic S, Stojkanovic G, Gjocaj C (2016). Hepato - Cephalic index as a predictor of Intrauterine Growth Restriction. Acta Inform Med.

[20] Castanys-Muñoz E, Kennedy K, Castañeda-Gutiérrez E, Forsyth S, Godfrey KM, Koletzko B, Ozanne SE, Rueda R, Schoemaker M, van der Beek EM, van Buuren S, Ong KK (2017). Systematic review indicates postnatal growth in term infants born small-for-gestational-age being associated with later neurocognitive and metabolic outcomes. Acta Paediatr.

[21] Nunes M, da Silva CH, Bosa VL, Bernardi JR, Werlang IC, Goldani MZ, NESCA Group (2017). Could a remarkable decrease in leptin and insulin levels from colostrum to mature milk contribute to early growth catch-up of SGA infants?. BMC Pregnancy Childbirth.

[22] de Brito ML, Nunes M, Bernardi JR, Bosa VL, Goldani MZ, da Silva CH (2017). Somatic growth in the first six months of life of infants exposed to maternal smoking in pregnancy. BMC Pediatr.

[23] Ben-Haroush A, Hadar E, Chen R, Hod M, Yogev Y (2009). Maternal obesity is a major risk factor for large-for-gestational-infants in pregnancies complicated by gestational diabetes. Arch Gynecol Obstet.

[24] Gaudet L, Ferraro ZM, Wen SW, Walker M (2014). Maternal obesity and occurrence of fetal macrosomia: a systematic review and meta-analysis. Biomed Res Int.

[25] Bakker H, Jaddoe VW (2011). Cardiovascular and metabolic influences of fetal smoke exposure. Eur J Epidemiol.

[26] Chelchowska M, Ambroszkiewicz J, Jablonka-Salach K, Gajewska J, Maciejewski TM, Bulska E, Laskowska-Klita T, Leibschang J (2013). Tobacco smoke exposure during pregnancy increases maternal blood lead levels affecting neonate birth weight. Biol Trace Elem Res.

[27] Oliveira C, Lins CP, Sá R, Netto HC, Bornia RG, Silva N, Junior JA (2006). Síndromes hipertensivas da gestação e repercussões perinatais. Rev Bras Saude Mater Infant.

[28] Eickmann SH, Lima M, Motta M, Romani S, Lira P (2006). Crescimento de nascidos a termo com peso baixo e adequado nos dois primeiros anos de vida. Rev Saúde Pública.

[29] Macdonald-Wallis C, Tilling K, Fraser A, Nelson SM, Lawlor DA (2013). Gestational weight gain as a risk factor for hypertensive disorders of pregnancy. Am J Obstet Gynecol.

[30] Rasmussen KM, Yaktine AL, National Research Council, Institute of Medicine, Board on Children, Youth, and Families, Food and Nutrition Board, Committee to Reexamine IOM Pregnancy Weight Guidelines (2009). Gestational weight gain as a risk factor for hypertensive disorders of pregnancy. Weight Gain During Pregnancy: Reexamining The Guidelines.

[31] Ferreira AP, Bernardi JR, Ferreira CF, Santos D, Santos KF, Pereira LW, Wainer M, Ferreira PK, Bosa V, Silva CH, Goldani MZ (2016). Fatores associados ao número de consultas pré-natais de mulheres tabagistas e não tabagistas atendidas em hospitais de porto alegre (RS), Brasil. Saúde em Redes.

[32] Galão A, Soder A, Gerhardt M, Faertes T, Kruger M, Pereira M, Borba CM SEER UFRGS.

[33] World Health Organization (2016). WHO Recommendations on Antenatal Care for a Positive Pregnancy Experience.

[34] IBGE, Coordenação de População e Indicadores Sociais (2015). Síntese de indicadores sociais: uma análise das condições de vida da população brasileira. Estudos e Pesquisas - Informação Demográfica e Socioeconômica.

[35] Buriol VC, Hirakata V, Goldani MZ, da Silva CH (2016). Temporal evolution of the risk factors associated with low birth weight rates in Brazilian capitals (1996-2011). Popul Health Metr.

[36] Schramm JM, Oliveira AF, Leite I, Valente JG, Gadelha AJ, Portela MC (2004). Epidemiological transition and the study of burden of disease in Brazil. Ciência & Saúde Coletiva.

[37] Barbieri MA, Bettiol H, Silva AA, Cardoso VC, Simões VM, Gutierrez MR, Castro JA, Vianna ES, Foss MC, Santos JE, Queiroz RG (2006). Health in early adulthood: the contribution of the 1978/79 Ribeirão Preto birth cohort. Braz J Med Biol Res.

[38] Mendes A, Sá DA, Miranda G, Lyra T, Tavares R (2012). [The public healthcare system in the context of Brazil's demographic transition: current and future demands]. Cad Saude Publica.

